# Exploring the use of digital technology to deliver healthcare services with explicit consideration of health inequalities in UK settings: A scoping review

**DOI:** 10.1177/20552076231185442

**Published:** 2023-06-29

**Authors:** Albert Farre, Mei Fang, Beth Hannah, Meiko Makita, Alison McFadden, Deborah Menezes, Andrea Rodriguez, Judith Sixsmith, Nicola M Gray

**Affiliations:** 1 School of Health Sciences, 3042 University of Dundee, Dundee, UK; 2 School of Humanities, Social Sciences and Law, 3042 University of Dundee, Dundee, UK; 3 The Urban Institute, 3120 Heriot Watt University, Edinburgh, UK; 4 School of Dentistry, 3042 University of Dundee, Dundee, UK

**Keywords:** Scoping review, digital technology, delivery of health care, health inequities, disadvantaged populations, United Kingdom

## Abstract

**Objective:**

To map and explore existing evidence on the use of digital technology to deliver healthcare services with explicit consideration of health inequalities in UK settings.

**Methods:**

We searched six bibliographic databases, and the National Health Service (NHS) websites of each UK nation (England, Scotland, Wales, Northern Ireland). Restrictions were applied on publication date (2013–2021) and publication language (English). Records were independently screened against eligibility criteria by pairs of reviewers from the team. Articles reporting relevant qualitative and/or quantitative research were included. Data were synthesised narratively.

**Results:**

Eleven articles, reporting data from nine interventions, were included. Articles reported findings from quantitative (n = 5), qualitative (n = 5), and mixed-methods (n = 1) studies. Study settings were mainly community based, with only one hospital based. Two interventions targeted service users, and seven interventions targeted healthcare providers. Two studies were explicitly and directly aimed at (and designed for) addressing health inequalities, with the remaining studies addressing them indirectly (e.g. study population can be classed as disadvantaged). Seven articles reported data on implementation outcomes (acceptability, appropriateness, and feasibility) and four articles reported data on effectiveness outcomes, with only one intervention demonstrating cost-effectiveness.

**Conclusions:**

It is not yet clear if digital health interventions/services in the UK work for those most at risk of health inequalities. The current evidence base is significantly underdeveloped, and research/intervention efforts have been largely driven by healthcare provider/system needs, rather than those of service users. Digital health interventions can help address health inequalities, but a range of barriers persist, alongside a potential for exacerbation of health inequalities.

## Introduction

The need for health systems worldwide to adapt to the restrictions brought about by the response to the COVID-19 pandemic has prompted an unprecedented acceleration of digital healthcare provision.^1^ These experiences have highlighted the benefits of embedding digital technologies in healthcare delivery, which have the potential to radically reshape the health services landscape if carried forward and built upon. However, the COVID-19 pandemic has also brought to the fore previously existing digital inequalities and exacerbated their health implications, particularly for those groups in society who were already most adversely affected by health inequalities, such as homeless people or people living with disabilities.^2–6^

The concept of digital inequalities differs from what is known as the digital divide, which focuses on people's ability to access digital technologies and the differences between those who are connected and those who are not. Conversely, digital inequality refers to how people of different backgrounds incorporate the digital world into their lives; how their digital and social contexts, their skills and their uses differ, and how the life outcomes associated with these differences vary.^7^ Therefore, the concept of digital inequality suggests that inequalities are not just a question of access to digital technologies but also stem from different ways of using and engaging with the digital world, and that inequalities would persist beyond issues of access.

Recent calls for digital technologies, or the digital ecosystem, to be recognised as a new determinant of health^8,9^ highlight the increasingly important interplay between digital inequalities and health outcomes, as well as the growing need to enable a more consistent understanding and measurement of these effects. Following the experiences during COVID-19 restrictions, the issue of whether future models of healthcare delivery should by default rely more heavily on digital technologies has received increasing attention in practice, research and policy debates.^10–12^ However, alongside acknowledging opportunities for improvement, clinicians and researchers have also warned about several important risks that such shift could bring,^11^ including risks to health equity and digital inclusion. In this context, it is therefore crucial to better understand the experiences and impact of digital health interventions for those most adversely affected by health inequalities. This understanding could help ensure that advances in digital health are not at the expense of widening health inequalities.

The aim of this scoping review was to map and explore the evidence available on the use of digital technology to deliver healthcare services with an explicit focus on or consideration of health inequalities in any healthcare setting within the UK.

The concept of ‘health inequalities’ can be comprehensively defined as the systematic, avoidable and unfair differences in health outcomes that can be observed between populations, between social groups within the same population, or as a gradient across a population ranked by social position.^13^

For the purpose of this review, we used the term ‘disadvantaged populations’ to refer to those most at risk of being adversely affected by the intersection of digital inequalities and health inequalities. This included population groups with protected characteristics in the UK by the Equality Act 2010^14^ (e.g. age, disability and race) as well as those generally recognised as being at increased risk of digital exclusion (e.g. those with a low income, homeless people and rural/remote communities).

Whilst previous reviews with a similar focus have been conducted globally, to our knowledge, this is the first review UK-based evidence on the use of digital technology to deliver healthcare services, with an explicit focus on or consideration of health inequalities at study population level, regardless of intervention type and healthcare setting. Previous reviews in the UK have adopted slightly different emphases, by either focusing on global evidence and excluding certain intervention types such as digital medical devices,^15^ or targeting specific healthcare settings such as general practice.^16^

## Methods

This scoping review was undertaken following the JBI scoping review methodology,^17,18^ and is reported in keeping with the PRISMA extension for Scoping Reviews (PRISMA-ScR).^19^

### Search strategy

We searched six biographic databases: MEDLINE, CINAHL Plus, PsycINFO, Web of Science (core collection), SCOPUS and The Cochrane Library (CDSR database). Searches were conducted in May 2021 using combinations of index terms and free text words relating to disadvantaged populations, digital health services and interventions, and health inequalities. A sample search strategy for MEDLINE is provided as supplementary material (Supplemental file 1). The National Health Service (NHS) website of each UK nation (England, Scotland, Wales and Northern Ireland) were hand searched. Reference lists of all included studies and relevant systematic reviews identified were scanned for eligible studies.

### Eligibility criteria and study selection

Studies of any research design were eligible for inclusion, including quantitative, qualitative and mixed-methods studies. Restrictions were applied on publication date (2013–2021) and publication language (English). Studies were included if they reported the use of digital technology to deliver health care services with an explicit focus on or consideration of health inequalities, in any context/setting within the UK. Studies were included if they reported the impact of digital health interventions on any disadvantaged populations in any setting within the UK. Studies were included if they focused on population groups with protected characteristics in the UK by the Equality Act 2010^14^ (e.g. age, disability and race) or who are generally recognised as being at increased risk of digital exclusion (e.g. those with a low income, homeless people, rural/remote communities).

Studies were excluded if they focused on population groups defined solely by a specific pre-existing medical condition (e.g. heart disease and mental ill health) which was not associated with a protected characteristic (e.g. age, disability and race). Studies were excluded if they were based in non-UK settings. Studies published in languages other than English were excluded. Studies published before 2013 were excluded to ensure that included interventions reflected the most up-to-date technologies. Review articles were excluded but used for reference checking to identify any additional, potentially relevant studies eligible for inclusion.

Titles, abstracts and relevant full texts were independently screened against the eligibility criteria by randomly assigned pairs of reviewers from the authorship group. Any disagreements between reviewers were resolved through discussion and consultation with a third reviewer or by consensus in wider team meetings.

### Data extraction

Data extraction was undertaken independently by randomly assigned pairs of reviewers from the authorship group using a piloted data extraction form. Any discrepancies between reviewers were resolved through team discussion and/or in consultation with a third reviewer.

### Data synthesis

Data were synthesised narratively, given that any pooled quantitative or qualitative analyses were considered inappropriate due to the broad range of interventions, study types and populations included in this review. Data extracted from included articles were categorised into three sets of categories to address our scoping review aims, based on (1) the types of interventions reported in the included studies; (2) the range of approaches employed to address health inequalities; and (3) the types of evaluations used to examine the impact of, and/or the perceptions about, the included interventions.

1. Intervention descriptions were coded and mapped against the World Health Organization's classification of digital health interventions,^20^ which provides a taxonomy of the different ways in which digital/mobile technologies are used to support health system needs and proposes a shared language to describe the uses of digital technology for healthcare provision.
2. Any approaches to address health inequalities reported in the included articles were coded and categorised according to whether they explicitly stated to address health inequalities or used any health inequalities conceptual frameworks at study design and/or at intervention development/implementation level.
3. Finally, study findings reported in the included articles were coded and categorised according to whether they evaluated implementation or effectiveness outcomes, and the findings summarised. Initial coding was undertaken by the lead author and then critically reviewed by the rest of the team. Further revision and refining of the coding and categorisation of included studies was then collectively undertaken by the team at regular meetings held throughout the data synthesis process.

## Results

**Figure 1.**
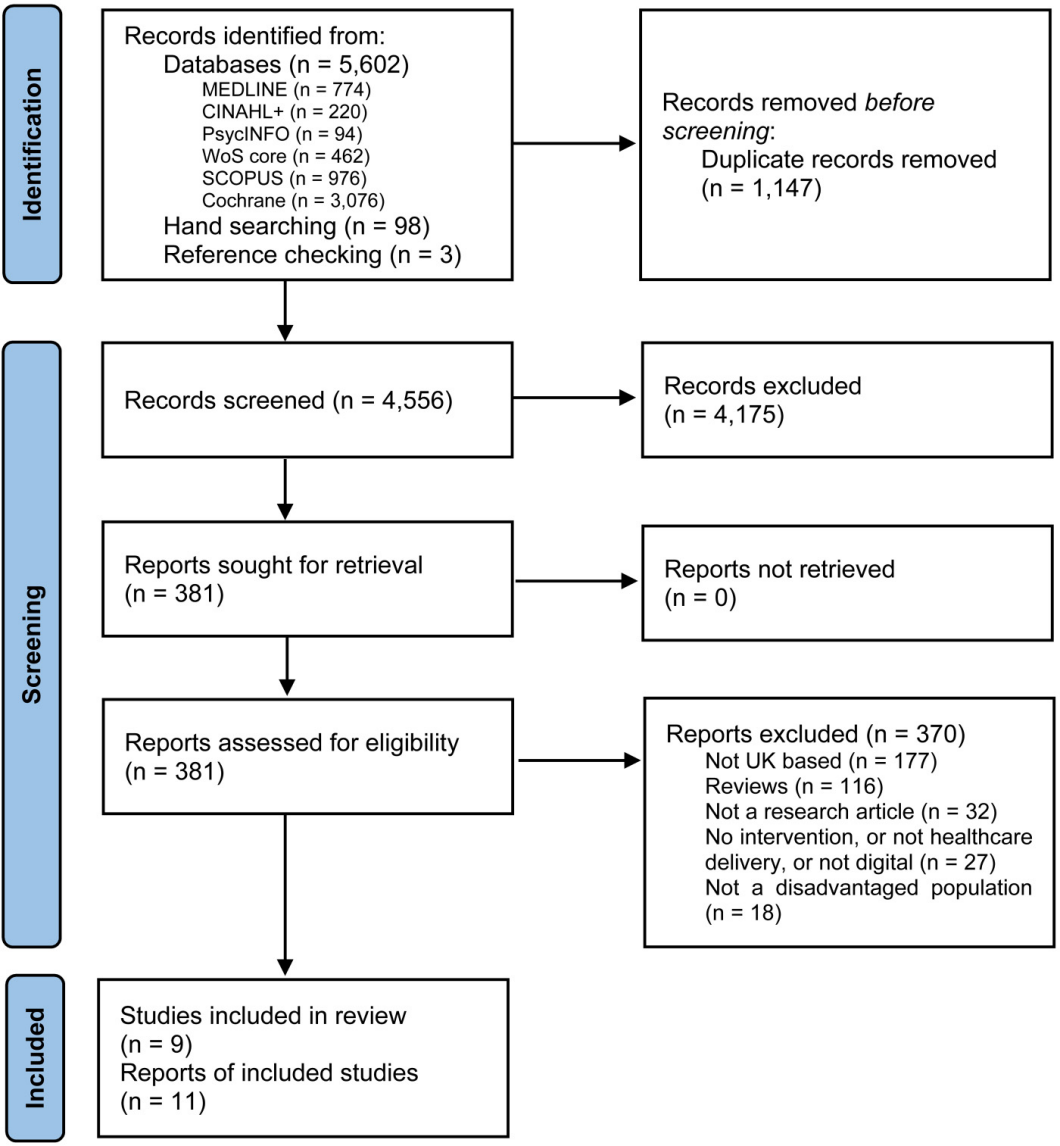
PRISMA flow diagram. From: Page et al.^42^

Systematic searches in bibliographic databases yielded 5602 records. A further set of 98 records were identified from hand searches in websites and grey literature sources, and 3 further records were identified through reference screening of relevant reviews identified. After deduplication, a final set of original records were assessed against the eligibility criteria. Title and abstract screening resulted in 381 records considered eligible or inconclusive. Full-text articles were then retrieved and assessed for eligibility, resulting in 11 articles reporting data from 9 interventions being included in the final synthesis (Figure 1).

### Summary of included studies

Five articles^21–25^ reported findings from quantitative studies (including three randomised controlled trials,^21,22,24^ one comparative before/after study^25^ and one cross-sectional study^23^); five more articles^26–30^ reported findings from qualitative studies (all interview studies); and one article^31^ reported findings from a mixed-methods study (combining quantitative routine data and qualitative interviews). Study settings were mainly community-based^22–24,26–31^ with only one intervention based in a hospital setting.^21^ A summary of key characteristics of included articles is presented in Table 1.

**Table 1.** Key characteristics of included articles.

Paper	Methodology	Study design	Setting	Population(s) at risk	Sample size	Intervention group sample size	Control group sample size	Intervention	Study aim
Akobeng (2015)	Quantitative	Randomised controlled trial	Hospital	Young people (aged 8–16 years) with inflammatory bowel disease (IBD) living at long distances from regional centres	86	44	42	Remote outpatient consultations	To evaluate effectiveness and cost consequences of the intervention compared to face-to-face consultations
Crombie (2018)	Quantitative	Randomised controlled trial	Community	Men from areas classified as being in the most disadvantaged quintile	825	411	414	Text messaging intervention	To evaluate effectiveness and cost-effectiveness of the intervention compared to attentional control text messages on general health topics
Hughes (2020)	Mixed-methods	Routine data (quant) and interviews (qual)	Community	Patients categorised as ‘high risk’ and ‘vulnerable’ by NHSE	781 (quant) 16 (qual)	-	-	Daily digitally supported conversations	To explore compliance with the intervention aims and assess the experiences of those involved
Latif (2019a)	Quantitative	Before/after questionnaire	Community	Marginalised or medically under-served groups	96	62	34	Digital (web-based) education	To evaluate whether a digital educational intervention could improve staff intention to engage with marginalised groups
Latif (2019b)	Qualitative	Interviews	Community		32	-	-		To investigate the impact of a novel digital educational intervention (e-learning resource) to improve access in one NHS-funded community pharmacy service known as ‘Medicines Use Reviews’ (MURs)
Rixon (2017)	Quantitative	Randomised controlled trial	Community	Older primary care patients with chronic obstructive pulmonary disease	447	275	172	Telehealth monitoring devices	To evaluate the effectiveness of the intervention on patients’ quality of life
Tuijt (2021)	Qualitative	Interviews	Community	Older people living with dementia and their carers	46	-	-	Remote primary care consultations	To understand the remote healthcare experiences of patients living with dementia and their family carers during the COVID-19 pandemic
Turnbull (2020)	Qualitative	Interviews	Community	People with type 2 diabetes (T2D) from low-income and ethnic minority populations	21	-	-	Digitally supported chronic condition self-care	To explore how adult users talk about their use of digital interventions for self-management of T2D by examining how they spoke about their identity in relation to their technology use and their illness
Turnbull (2021)	Qualitative	Interviews	Community			-	-		To explore how and why people with T2D access and use digital health technologies to help them manage their condition and how experiences vary between individuals and social groups
Vereenooghe (2017)	Qualitative	Interviews	Community	People with an intellectual disability	6	-	-	Digital delivery of psychological therapy	To identify the (a) functions and benefits, (b) challenges and barriers, and (c) required design features of computers in therapy for people with intellectual disability
Walters (2017)	Quantitative	Feasibility study, questionnaires	Community	Older people	454	-	-	Multi-dimensional Risk Appraisal for Older people (MRA-O) system	To test the feasibility and costs of using HRA-O and SWISH tools combined into a MRA-O

### Intervention types and functionalities

Two studies^24,28,29^ related to interventions targeting service users, and seven studies^21–23,26,27,30,31^ related to interventions targeting healthcare providers.

#### Interventions for service users

- *Targeted service user communication:* One study^24^ developed and evaluated an intervention using mobile phones to deliver a series of interactive text messages to reducing binge drinking in disadvantaged men from areas of high deprivation. The intervention's primary digital functionality was to transmit targeted alerts and reminders to service users, relying on information systems from primary care (general practice registers) and a Short Message Service system.
- *Personal health tracking:* One study^28,29^ explored users’ experiences of a set of digital health interventions whose main digital functionality was to enable self-monitoring of health/diagnostic data by service users. The study focused on examining the experiences of those in lower-income neighbourhoods and Black, Asian, and Minority Ethnic groups. The range of interventions used by participants included wearable trackers, mobile apps and web-based tools to enable type 2 diabetes patients to develop self-care expertise, all of them being user-owned and user-managed devices or applications either supplied by health providers or purchased privately.

#### Interventions for healthcare providers

- *Healthcare provider decision support:* One study^23^ adapted and feasibility tested a computer-aided Multi-dimensional Risk Appraisal for Older people (MRA-O) system, comprising: (1) postal questionnaire including health, lifestyle, social and environmental domains; (2) software system generating a personalised feedback report with advice on health and wellbeing; (3) follow-up of people with new concerning or complex needs by general practitioners or practice nurses. The intervention's main digital functionality was to enable healthcare providers to screen service users by risk or other health status, relying on linking the intervention with general practices health records. Key health system challenges addressed by this intervention related primarily to the ‘accountability’ domain, as well as the ‘information’ provision and service ‘utilisation’ domains.
- *Telemedicine interventions:* Four interventions^21,26,30,31^ focused on delivering consultations between remote service users and healthcare providers: The first study^21^ evaluated an intervention targeting young people with inflammatory bowel disease (IBD) living at long distances from a regional paediatric referral centre, using telephone-based outpatient consultations delivered by gastro-enterology doctors; the second study^26^ examined the experiences of online provision of psychological therapies for people with intellectual disabilities; a third study^31^ evaluated an intervention based on telephone conversations delivered by medical students for populations categorised as ‘high risk’ and ‘vulnerable’ to COVID-19 infection in a rural primary care setting; and the fourth study^30^ examined the implications of remote primary care consultations (mainly telephone-based but also including video consultations) for older people living with dementia and/or their carers during the COVID-19 pandemic.

Another telemedicine intervention^22^ focused on enabling remote monitoring of service user health/diagnostic data by healthcare providers. The intervention used peripheral devices for older patients with chronic obstructive pulmonary disease (COPD) (pulse oximeter with/without blood pressure monitor, weight scales and additional peripherals depending on clinical need) which were attached to a home monitoring system to allow questions and educational messages to be transmitted to participants.

- *Healthcare provider training:* One study^25,27^ developed and evaluated the impact of a co-produced digital education intervention (e-learning resource comprising videos, interactivities and self-assessments) for community pharmacy professionals, including: discovering and understanding underserved communities; exploring the medicine experiences and needs of patients who are underserved; and effectively interacting and engaging patients who are underserved. The intervention's main digital functionality was to provide training content to healthcare providers, relying on a self-developed learning and training system.

### Strategies used to address health inequalities

Table 2 summarises the strategies to address health inequalities identified in the included articles.

**Table 2.** Strategies used to address health inequalities.

Strategies to address health inequalities reported in included articles		Included studies								
		Akobeng (2015)	Crombie (2018)	Hughes (2020)	Latif (2019a, 2019b)	Rixon (2017)	Tuijt (2021)	Turnbull (2020, 2021)	Vereenooghe (2017)	Walters (2017)
At study design level	Study aims/objectives or tailored study components (e.g. sampling) explicitly addressing health inequalities				⬤			⬤		
	Use of any health inequalities concepts/frameworks to inform data collection/analysis methods		⬤		⬤	⬤	⬤	⬤		
	Targeted study population that can be classed as ‘disadvantaged’ or at risk of health inequalities	⬤	⬤	⬤	⬤	⬤	⬤	⬤	⬤	⬤
At intervention level	Intervention aims/objectives or tailored intervention components/features explicitly addressing health inequalities				⬤					
	Use of any health inequalities concepts/frameworks to inform intervention development/adaptations/delivery				⬤					
	Intervention specifically designed/tailored to a population that can be classed as ‘disadvantaged’ or at risk of health inequalities		⬤	⬤	⬤					⬤

Only two studies^25,27–29^ were identified to be directly aimed at, and designed for, addressing health inequalities; however, only one of them^25,27^ related to an intervention also explicitly aimed at, and designed for, addressing health inequalities.

Three further studies^22,24,30^ addressed health inequalities by embedding the use of health inequalities concepts/frameworks to inform their methods (e.g. use of Index of Multiple Deprivation for sampling); however, only one of these^24^ related to an intervention specifically designed/tailored to a population that can be classed as ‘disadvantaged’ or at risk of health inequalities.

The remaining four studies^21,23,26,31^ addressed health inequalities indirectly (i.e. without explicitly claiming to do so) by targeting study populations that could be classed as ‘disadvantaged’ or at risk of health inequalities, with only two studies^23,31^ relating to interventions with components specifically tailored to those populations. It is however important to note that, the two studies^21,26^ that did not report any specifically tailored components were both to deliver remote consultations assumed to be patient-centred in terms of content, which was by definition tailored by clinicians at the point of delivery, and therefore any further tailoring may have been considered unnecessary by study/intervention teams.

Overall, the interventions identified were largely generic (five out of nine). Only one intervention^25,27^ was explicitly aimed at, and designed for, addressing health inequalities. Three further interventions^23,24,31^ did not explicitly claim to be addressing health inequalities, but were specifically designed/tailored to a population that can be classed as ‘disadvantaged’ or at risk of health inequalities.

### Intervention evaluations: implementation outcomes

Digital delivery of psychological therapy was reported as having the potential to improve therapy engagement and experiences for people with an intellectual disability.^26^ Digital technologies can help this population overcome in-session communication difficulties and practise skills at home. On-screen pictures, interactive games, symbols, sign language and touch-screens are key design features to help engagement. Implementation barriers include clinician-reported difficulties relating to their own capacity/capability to access and use digital technology and fitting it into their own defined roles.

Digital educational interventions were found to increase pharmacy professionals’ awareness and motivation to engage with marginalised groups, but key structural implementation barriers (e.g. perceived excessive workload) were found to often hinder translation into practice.^27^ If supported and promoted effectively by policy makers and employers, digital educational interventions have the potential to enable and facilitate ways for pharmacy professionals to better engage with marginalised groups and bring about improvements in patients’ health and medicines management.

Included studies^28,29^ suggest that digital interventions to help self-manage long-term conditions can exacerbate health inequalities if experiences of patients from low-income and ethnically diverse communities are not well addressed in intervention development and implementation. Low levels of digital skills and high cost of digital health interventions can also create barriers to access and use of digital interventions to support self-management of long-term conditions.^29^ Personal social networks and social status can be leveraged to overcome some of these challenges, therefore, health inequalities can also be exacerbated when those resources are not present in people's lives.

Remote primary care telephone-based consultations may not be well-suited to address the needs and preferences of people living with dementia and their carers,^30^ who reported to prefer face-to-face contact. Furthermore, digital and language barriers were reported to prevent older people from accessing virtual services. Remote consultations may not be a good medium for identifying and addressing new problems for people living with dementia and their carers and may limit health providers’ ability to tailor services to individual needs for these populations. However, more targeted interventions delivered as an add-on to routine primary care, such as daily calls to patients categorised as ‘high risk’ and ‘vulnerable’,^31^ have the potential to provide additional practical and psychological benefits for these populations, including strengthening their links with community voluntary groups, as well as provide additional opportunities for medical education in primary care settings.

A computer-aided risk appraisal system was found to be feasible to implement for general practices, yielding useful information about health and social problems, and enabling identification of some individual needs.^23^ However, participation rates in the feasibility study were particularly low for the oldest old, the poorest and ethnic minority groups, which suggests that this type of intervention may increase inequalities in access. Implementation at scale of this approach would require work to address potential exacerbation of health inequalities.

Overall, seven articles^23,26–31^ reported data on implementation outcomes, with reports primarily relating to issues of acceptability, appropriateness, and feasibility of digital health interventions. Of the seven articles, five reported on interventions for healthcare providers^23,26,27,30,31^ and two reported on interventions for service users.^28,29^

### Intervention evaluations: effectiveness outcomes

A randomised controlled trial and economic evaluation^21^ found remote consultations to be a cost-effective alternative to face-to-face consultations for the routine outpatient follow-up of children and adolescents with IBD living at long distances from regional centres. No inferiority of telephone-based versus face-to-face consultation was found in relation to improvements in quality-of-life scores (estimated treatment effect in favour of the telephone consultation group was 5.7 points, 95% confidence interval (CI) −2.9 to 14.3; p = 0.19). Telephone consultation reduced consultation time, with an estimated reduction (95% CI) of 4.3 (2.8 to 5.7) minutes in consultation times (p < 0.001); and NHS costs, with a difference of £15.71 (95% CI 11.8–19.6; p < 0.001) per patient consultation.

Another randomised controlled trial^22^ showed that telehealth monitoring devices with educational messages for patients with COPD did not reduce quality-of-life nor increase psychological distress. There were small effects (p < 0.05) for improved emotional functioning and mastery over COPD at long-term follow-up (12 months) but not at short-term follow-up (4 months).

An uncontrolled, paired before-and-after survey^25^ found an improving trend in pharmacy professionals’ intention to engage with marginalised groups in the short-term after participating in a digital educational intervention, with a significant increase (0.44; 95% CI 0.11 to 0.76, p = 0.009) identified in one out of five behaviours change intention constructs measured (participants’ beliefs about capabilities). However, no significant change was detected in the numbers of patients being offered a medication review.

Finally, a randomised controlled trial^24^ showed that text messages to have had a modest, statistically non-significant effect on the proportion of disadvantaged men binge drinking (consuming > 8 units of alcohol) on ≥3 occasions in the previous 28 days, assessed at 12-month follow-up (odds ratio 0.79, 95% confidence interval (CI) 0.57 to 1.08). However, the trial demonstrated that it is possible to recruit and retain large numbers of disadvantaged men in a research study.

Overall, four articles^21,22,24,25^ reported data on effectiveness, including the following primary outcomes: quality of life,^21,22^ intention to engage with marginalised groups,^25^ and proportion of disadvantaged men binge drinking.^24^ Three studies^21,22,25^ evaluated interventions for healthcare providers, and one study^24^ evaluated an intervention for service users.

## Discussion

This scoping review sought to map and explore the evidence available on the use of digital technology to deliver healthcare services with an explicit focus on or consideration of health inequalities in UK healthcare settings. Our findings suggest that the current evidence base in this area is significantly underdeveloped, with only eleven articles (reporting on nine interventions/services) identified between 2013 and 2021.

Within this, we found that evidence on effectiveness and health outcomes for those most at risk of health inequalities in the UK is particularly limited. Only four studies^21,22,24,25^ were identified that contributed data on effectiveness to our synthesis, of which only one study^21^ demonstrated cost-effectiveness. Therefore, it is not yet clear if digital health services in the UK work for those most at risk of health inequalities. This is particularly concerning in a potentially radically changing health services landscape, building on service delivery models developed to support COVID-19 restrictions.^10–12^ In this context, some interventions previously used only as add-ons to routine care (e.g. remote consultations to address geographical inaccessibility of services in rural and remote populations) could now become part of mainstream service delivery. This, in turn, makes the need to better understand the impact of digital health interventions on those most at risk of health inequalities a more fundamental, pressing issue.

The body of evidence identified in this review suggests that research so far has been largely driven by healthcare provider's needs rather than those of service users from populations most at risk of health inequalities. Similarly, the evidence on implementation outcomes identified in this review suggests that digital health interventions remain better aligned with the needs of healthcare providers than those of service users from more disadvantaged populations.

Although the evidence we identified also suggests that digital health interventions can offer promising new avenues to address health inequalities,^15^ some significant structural barriers were reported to be at play across most intervention domains as well as the potential for very important unintended impacts such as the exacerbation of health inequalities.^2–5^

Taken together, these findings highlight the importance for future digital health research and interventions/service development efforts to meaningfully engage with disadvantaged populations, for example, drawing on philosophies such as co-production and co-design, which acknowledge that services users are best placed to advise on how services can be made more accessible to them, while also appreciating the views and needs of those responsible for service delivery.^32–34^

### Recommendations for practice, research and policy

To avoid digital health services inadvertently widening health inequalities, investments in inclusion should be embedded into any investments in digital health.^2,15^ In this context, the role of implementation research in this area could be of particular importance to advance the understanding of digital health inequalities and enable successful integration of health equity into implementation models.^35^ Likewise, research and interventions addressing key issues that could mitigate against the emergence of digital health inequalities can be of particular importance in this context.^5,6,15^ For example, interventions to facilitate access to connected devices and interventions to increase digital literacy, health literacy or digital health literacy, which for some time have shown potential to improve outcomes for populations most at risk of health inequalities.^36–38^ Digital-based approaches to support digital health literacy in disadvantaged populations present new opportunities that can complement traditional non-digital strategies, but existing evidence in this area is still sparse.^39^ Alongside this, it is important to acknowledge the need to better understand and monitor changing patterns in the use of digital technologies and social media platforms as health support resources,^15,40,41^ which can in turn change the nature of digital health inequalities. This is in line with calls to consider digital technologies as a new determinant of health.^8,9^

To enable advances in the understanding of digital health inequalities, more theoretically informed and purposely designed interventions and studies focusing on digital health inequalities are needed. We only identified one intervention^25,27^ and two studies^25,27–29^ being directly aimed at, and designed for, addressing digital health inequalities. The remaining interventions and studies only addressed health inequalities indirectly, by either embedding key concepts/frameworks into their methods (e.g. use of Index of Multiple Deprivation for sampling) or by targeting study populations that could be classed as ‘disadvantaged’ or at risk of health inequalities. These are important gaps in the evidence available in UK settings, which is much needed to better understand the implications of digital health interventions for those most at risk of health inequalities.

Alongside this, the broad range of intervention types and functionalities identified in this review should also be considered in future research in this area, particularly in terms of better understanding how digital health inequalities align with existing health-system and information-system challenges, to inform governance and organisational changes required to enable sustainable digital health inclusion efforts at scale.

### Strengths and limitations

To our knowledge, this is the first review to bring together and examine primary quantitative and qualitative research relevant to the use of digital technology to deliver healthcare services with an explicit focus on or consideration of health inequalities, in any healthcare setting within the UK. There were some limitations in our search strategy, including limits placed in publication language (only articles in English were included) and publication date (2013–2021). Also, our focus on studies examining the impact of digital interventions on disadvantaged populations carries the limitation that any studies evaluating a health-inequalities-tailored digital health intervention using a general population sample may have been missed. Follow-up review work examining this body of evidence could make an important contribution and inform advances to help achieve equitable health goals in the UK.

## Conclusions

It is not yet clear if digital health interventions/services in the UK work for those most at risk of health inequalities. Our findings suggest that the current evidence base in this area is significantly underdeveloped, with only four studies identified that contributed data on effectiveness to our synthesis, of which only one study demonstrated cost-effectiveness.

So far, research and intervention/service development efforts in this area have been largely driven by healthcare provider/system needs, rather than those of service users. A key priority for future UK-based digital health research/interventions should be to meaningfully engage with disadvantaged populations to inform their approaches.

Although the current evidence suggests that digital health interventions can offer promising new avenues to address health inequalities, it also suggests that a range of barriers persist, alongside a potential for exacerbation of health inequalities. To avoid digital health services inadvertently widening health inequalities, investments in inclusion should be integral to any investments in digital health. To successfully enable this, it is paramount to address another important gap identified in this review, the scarcity of theoretically informed digital health research/interventions purposely designed to address health inequalities as an integral part of their goals. These are fundamental developments that should become normalised if we are to achieve equitable digital healthcare provision in the UK.

## Supplemental Material

sj-docx-1-dhj-10.1177_20552076231185442 - Supplemental material for Exploring the use of digital technology to deliver healthcare services with explicit consideration of health inequalities in UK settings: A scoping review Click here for additional data file. Supplemental material, sj-docx-1-dhj-10.1177_20552076231185442 for Exploring the use of digital technology to deliver healthcare services with explicit consideration of health inequalities in UK settings: A scoping review by Albert Farre, Mei Fang, Beth Hannah, Meiko Makita, Alison McFadden, Deborah Menezes, Andrea Rodriguez, Judith Sixsmith and Nicola M Gray in DIGITAL HEALTH
