# Supplementary material for: Exploring the use of digital technology to deliver healthcare services with explicit consideration of health inequalities in UK settings: A scoping review
Source: Digit Health. 2023 Jun 29;9:20552076231185442. doi: 10.1177/20552076231185442 (PMC10328001; doi:10.1177/20552076231185442)
Supplement: sj-docx-1-dhj-10.1177_20552076231185442 - Supplemental material for Exploring the use of digital technology to deliver healthcare services with explicit consideration of health inequalities in UK settings: A scoping review [file sj-docx-1-dhj-10.1177_20552076231185442.docx]

**Supplementary file 1 - Search strategy for MEDLINE**

| Filters applied | (English[Language]) AND ("2013/01/01"[Date - Publication] : "2022"[Date - Publication]) |  |
| --- | --- | --- |
| 1 | ("National Health Service" OR "NHS" OR healthcare OR "health care" OR "health service*" OR "health service design" OR "health service delivery" [Title/Abstract]) | 340,300 |
| 2 | (intervention* OR initiative* OR program* OR platform* OR implement* OR improv* [Title/Abstract]) | 2,232,330 |
| 3 | (vulnerable OR sensitive OR disadvantaged OR underserved OR marginali* OR deprived OR excluded OR stigmati* OR discriminated [Title/Abstract]) | 400,810 |
| 4 | (eHealth OR Telehealth OR mHealth OR "mobile health" OR "web-based" OR "digital" OR telecare OR app OR technology OR computer OR tablet OR "mobile phone" or smartphone OR internet [Title/Abstract]) | 1,859,368 |
| 5 | ("divide" OR "gap" OR inequalit* OR equalit* OR disparit* OR "determinants" OR "exclusion" OR "inclusion" OR "equity" OR "inequity" [Title/Abstract]) | 416,107 |
| 6 | 1 AND 2 AND 3 AND 4 AND 5 | 774 |
